# Geographic body size variation of a tropical anuran: effects of water deficit and precipitation seasonality on Asian common toad from southern Asia

**DOI:** 10.1186/s12862-019-1531-z

**Published:** 2019-11-09

**Authors:** Cheng Guo, Shuai Gao, Ali Krzton, Long Zhang

**Affiliations:** 1 Department of Zoology, College of Life Science and Technology, Central South University of Forestry and Technology, Changsha, 410004 Hunan China; 2 Department of Research and Instruction, RBD Library, Auburn University, Auburn, AL 36849 USA

**Keywords:** Bergmann’s rule, *Duttaphrynus melanostictus*, Precipitation seasonality, Water-energy conservation hypothesis, Water deficit

## Abstract

**Background:**

Two previous studies on interspecific body size variation of anurans found that the key drivers of variation are the species’ lifestyles and the environments that they live in. To examine whether those findings apply at the intraspecific level, we conducted a study of the Asian common toad (*Duttaphrynus melanostictus*), a terrestrial anuran distributed in tropical regions. The body size of toads from 15 locations, covering the majority of their geographic range, and local environmental data were summarized from published literature. We used a model selection process based on an information-theoretic approach to examine the relationship between toad body size and those environmental parameters.

**Results:**

We found a positive correlation between the body size of the Asian common toad and the water deficit gradient, but no linkage between body size and temperature-related parameters. Furthermore, there was a positive correlation between the seasonality of precipitation and body size of females from different sampled populations.

**Conclusions:**

As a terrestrial anuran, the Asian common toad should experience greater pressure from environmental fluctuations than aquatic species. It is mainly distributed in tropical regions where temperatures are generally warm and stable, but water availability fluctuates. Therefore, while thermal gradients are not strong enough to generate selection pressure on body size, the moisture gradient is strong enough to select for larger size in both males and females in dryer regions. Larger body size supports more efficient water conservation, a pattern in accordance with the prediction that lifestyles of different species and their local habitats determine the relationship between body size and environment. In addition, larger females occur in regions with greater seasonality in precipitation, which may happen because larger females can afford greater reproductive output in a limited reproductive season.

## Background

**Fig. 1:**
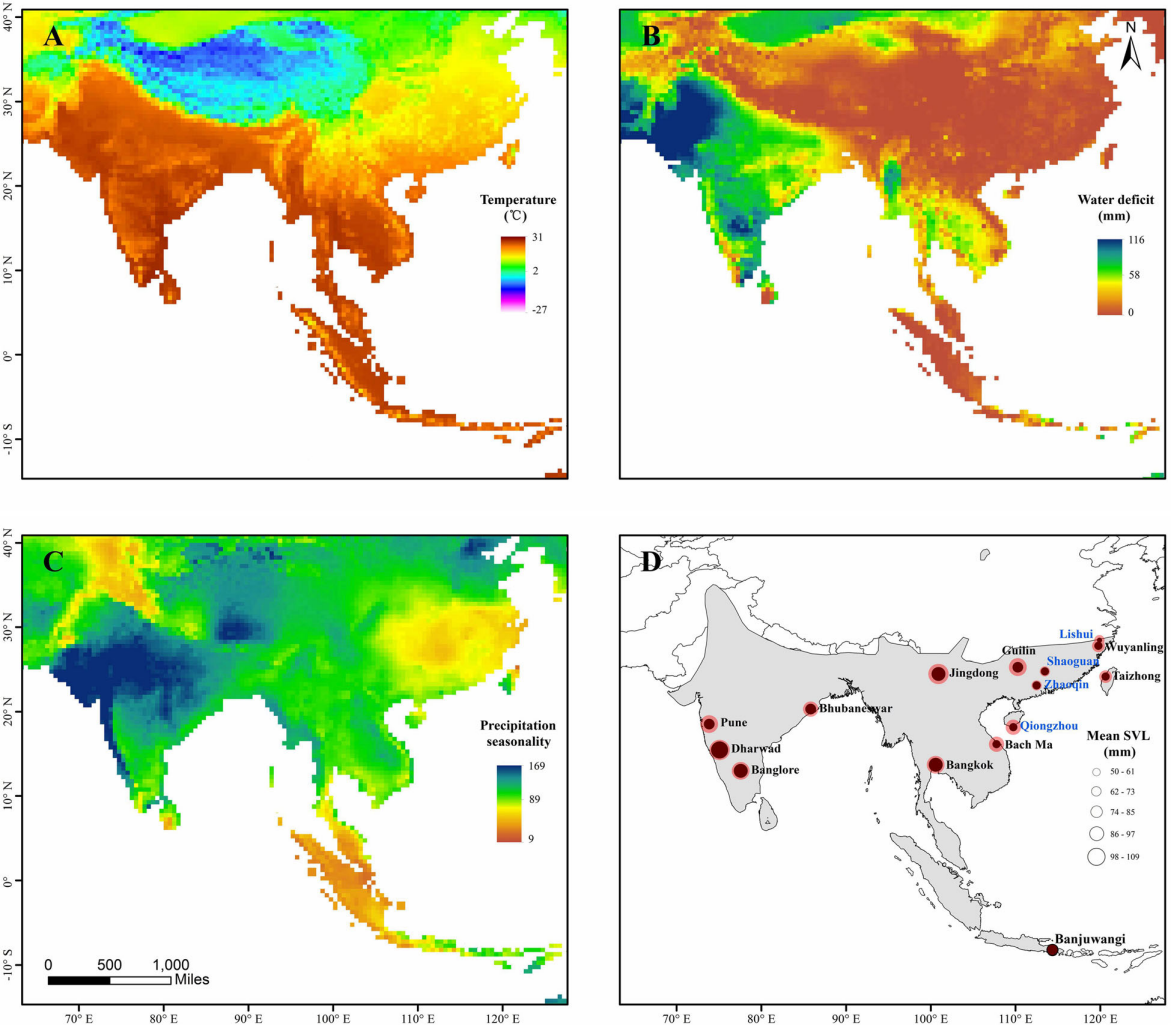
Body size variation of sampled populations along the environmental gradients. Climatic layers are (**a**) temperature, (**b**) water deficit and (**c**) precipitation seasonality [36–38]. Grey areas  in (**d**) are main distribution range of the toad [39]. Cycle size represent spatial pattern of mean SVL for the toad. Mean body size of males is indicated by black cycles, and females are indicated by red cycles. Locations showed in blue are sampled localities of a previous study [40]

Body size is an important life history trait that influences many aspects of an individual’s biology [1], and identifying the forces (i.e. environmental gradients, interaction with sympatric species) that influence geographic body size variation across different species has implications for understanding how animals adapt to different abiotic and biotic environments [2–6]. A well-known clinal pattern of body size variation is Bergmann’s rule, which describes the tendency for body size to increase with lower temperatures or higher latitudes, because body size evolves under selective pressure from ambient temperature; being larger helps maintain body heat in cold environments (the heat conservation hypothesis [7, 8]). The majority of endothermic vertebrates show a Bergmann’s cline at the intraspecific [9] as well as interspecific [10, 11] level, and environmental temperature gradient is a decisive abiotic factor in body size diversification [7–11]. For insight into the issue, it was found that some anurans also show a Bergmann’s cline, with environmental temperature acting as the primary determinant of geographic size variation like in endotherms at both interspecific and intraspecific levels [12, 13]. Specially, many intraspecific investigations demonstrated that some life history traits diversify, interact and trade off under the temperature gradients and represent in the form of body size variation along the gradients [3, 4, 14–20]. Generally, a bigger size due to longer longevity in colder environments also facilitates heat conservation for many anuran species [3, 4, 16]. Nevertheless, there are also many species show a reversed cline [17–19], while some others show no geographic trend [20–22] even some life history traits show some general clines [17, 19, 21, 22]. Furthermore, aside from temperature as proposed by the heat conservation hypothesis, environmental factors such as water deficits and seasonality of temperature are emphasized in other hypotheses concerning body size variation in ectotherms, e.g. the water availability hypothesis [23]; the hibernation hypothesis [24] and the heat balance hypothesis [13]. Moreover, because males and females may suffer different pressures from the same gradient, some species show sex-specific relationships between body size and environmental/geographic gradients [17, 20, 21, 25, 26]. The question of why there are such disparate trends among anurans remains unanswered. Studies of other clades suggest that these different relationships and new relevant factors emerge due to species-specific traits (i.e. habitat preference or thermal niche) [2, 27, 28] and the particular characteristics of each sex [29]. As for anurans, the body size of anurans in Europe and North America are negatively correlated with and environmental energy gradients in accordance with most previous findings [3, 4, 7–13]. Nevertheless, anurans from the Brazilian Cerrado in South America showed a positive correlation between body size and water deficit, a measurement of environmental dryness [30]. Those findings indicate that body size in anurans is strongly affected by environmental background: in regions where temperature fluctuates more, but precipitation fluctuates less (e.g., Holarctic), the major forces are thermal conditions, but in regions where temperature fluctuates less than precipitation (e.g., Neotropics), precipitation becomes the limiting factor [30]. Based on these findings, Olalla-Tárraga et al. (2009) proposed the water-energy conservation hypothesis, which posits that larger bodies allow greater heat conservation in cold macroclimates and greater water conservation in dry tropical areas [30]. A subsequent study on Chinese anurans showed that terrestrial anurans were generally larger in colder regions because they face temperature stress directly from the ambient environment, and becoming larger helps to conserve body heat. However, size of aquatic species did not change along thermal gradients. This could be due to the buffering effect of water, which reduces the pressures from macroenvironmental fluctuation. In other words, the relatively stable environmental gradients aquatic anurans experience are not strong enough to drive the evolution of their body size. Ecological traits (referring, for example, to the habitat preference of different species, i.e. terrestrial/aquatic/arboreal) of different clades also play an important role in the body size-environment relationship [31]. Furthermore, a recent interspecific study on new world anurans further indicated that clades with different habitat preferences suffer different selective pressures [32]. These studies of anurans on the American continents and in China focused on interspecific variation in body size [30–32]. However, the mechanisms that drive interspecific variation in body size should also apply to intraspecific variation [33, 34]. At present, studies considering both the ecological traits and environmental background within single anuran species are rare. A follow-up study found that body size in Darwin’s frogs (*Rhinoderma darwinii*) is positively correlated with the magnitude of seasonal temperature differences. Such a linkage may arise due to metabolic rate is reduced further and longer during colder, longer winters, leading to decreased energy depletion during hibernation, improved survival and increased longevity (and hence growth) (a mechanism termed the hibernation hypothesis) [24]. Another study on male lesser treefrogs (*Dendropsophus minutus*) found that body size is positively linked with increased seasonality of precipitation, in accordance with the predictions of the water-energy conservation hypothesis [35]. Nevertheless, neither study addressed the role of ecological traits and the environmental background in the patterns of variation observed within the focal species. The patterns discovered may owe to direct selective pressures from ambient environmental gradients (terrestrial and arboreal) in both species, but selective pressures probably differ because they live in different regions (temperate forests vs. tropical regions) [24, 35]. More work on other species in other regions is needed to clarify the issue. The Asian common toad (*Duttaphrynus melanostictus*) is a terrestrial anuran distributed widely throughout Southeast Asia and as far west as India and Pakistan (Fig. 1d) [39]. As a terrestrial species, it spends the majority of its life on land and may suffer more extremes of ambient environmental conditions than aquatic species [31, 41–43]. Its body size may evolve differentially due to localized environmental pressures, with populations from different locations exhibiting a size cline along some environmental gradients. The Asian common toad faces macroenvironmental conditions similar to those of the Brazilian Cerrado [30], with warm and stable temperatures that do not fluctuate much across its geographic range (inferred by overlapping its distribution map with environmental data downloaded from WorldClim [36] in ARCGIS 10.0). However, precipitation conditions across its distribution range are characteristically variable, fluctuating across both space and time [36, 39]. As a result, populations of Asian common toads in different locations may experience more environmental pressure from precipitation than from temperature [30]. The patchy environmental conditions that Asian common toads face allow us to test the validity of the water-energy conservation hypothesis within a single species. A previous study conducted on body size variation in the Asian common toad within a small geographic range (Fig. 1d) found that this species exhibited a reversed Bergmann’s cline [40]. However, sampling a relatively small proportion of territory in a species with a broad geographic range may give an incomplete picture of intraspecific variation [44]. In the present study, we summarized data covering the majority of the Asian common toad’s distribution to explore the relationship between body size and environmental predictors to gain insight into intraspecific responses to variations in environmental background. We hypothesized that toads would be larger in regions where the environmental water deficit is high, but that there would be no relationship between body size and thermal gradients [30]

## Results

### Species and environmental data

The dataset was summarized from the literature, which included 15 populations covering the majority of the distribution of the Asian common toad. The mean body size of males ranged from 50.8 mm to 90.6 mm, and the mean body size of females ranged from 58.0 mm to 107.1 mm (Fig. 1d; Additional file 1: Table S1). The annual mean temperature experienced by each population ranged from 15.8 °C to 28.1 °C; the water deficit level ranged from 0.46 mm to 66.28 mm; and the precipitation seasonality ranged from 49 to 104 (Fig. 1a-c; Additional file 1: Table S2). The mean snout-vent length value of both males and females were spatially autocorrelated (Moran’s *I* = 0.44, *P* = 0.001; Moran’s *I* = 0.28, *P* = 0.026 separately), indicating that nearby populations have similar body sizes.

### Correlation between body size and environmental gradients

**Fig. 2:**
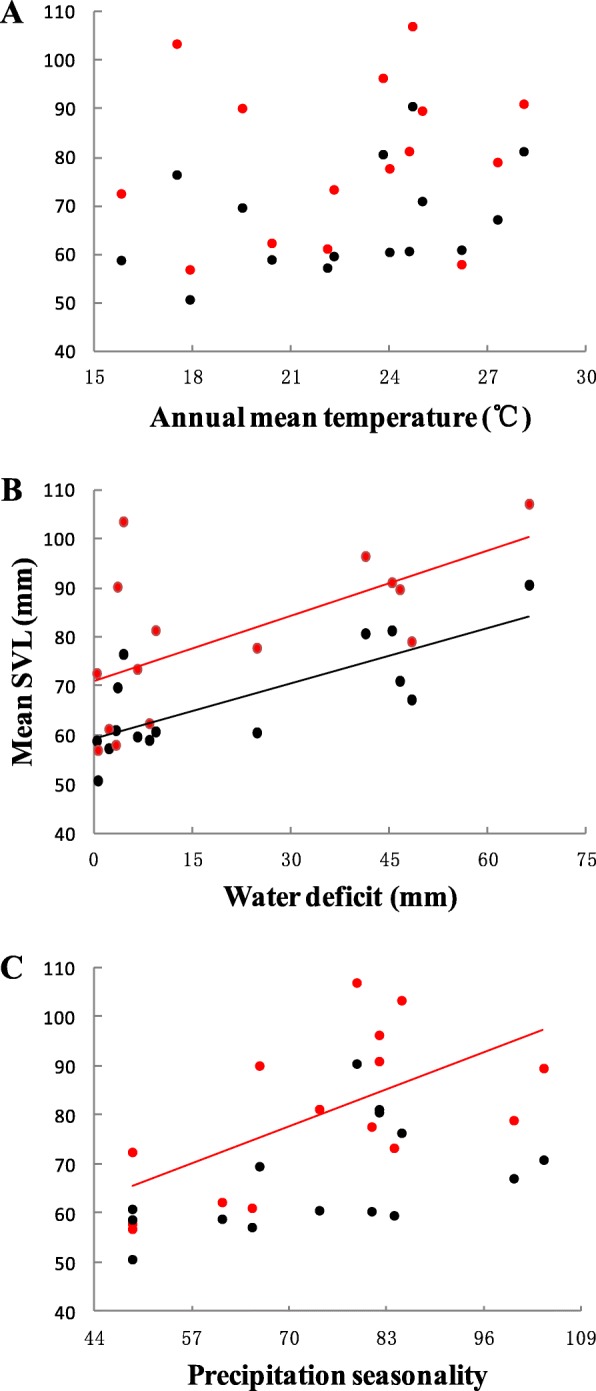
Mean body size of male and female Asian common toad as a function of annual mean temperature (**a**), water deficit (**b**) and precipitation seasonality (**c**). Males are indicated by black circles and black lines, and females are indicated by red circles and red lines

When sex differences were considered, of the 32 models constructed, results showed that the mean body size of male Asian common toad was unaffected by environmental temperature gradients but was influenced by environmental dryness level (Table 1; Fig. 2a and b). The best model contained water deficit only, with mean male body size positively and significantly correlated with water deficit (β = 0.369 ± 0.088, *P* = 0.001; Fig. 2b). This factor alone explained 54.2% of the body size variation in males; no linkage with other predictors was detected (Table 1). Similarly, among females, mean female body size was unaffected by environmental temperature gradients (Table 1; Fig. 2a), and water deficit alone was the second-best-fit model, explaining 33.1% of the variation (β = 0.439 ± 0.156, *P* = 0.015; Fig. 2b). In contrast to the males, however, the body size of female Asian common toads was also influenced by the seasonality of precipitation, as female body size was positively and significantly correlated with environmental precipitation seasonality. For females, the best model contained precipitation seasonality only, which alone explained 33.8% of variation in body size (β = 0.576 ± 0.202, *P* = 0.014; Fig. 2c). The residuals of the best models were not spatially autocorrelated (Monte Carlo permutation test, all *P* > 0.1 for mean and both sexes), indicating that spatial autocorrelation did not bias our results. Sex differences were ignored in the analysis in some previous studies. When we combined the data for both sexes, of the 32 models constructed, the model selection process indicated that body size was unaffected by either environmental temperature gradients or by actual evapotranspiration, as in the sex-specific analysis, but the variation also could not be explained by precipitation seasonality (Table 1). The mean body size of Asian common toads was affected by water availability only, with a positive correlation between mean body size and water deficit (β = 0.362 ± 0.121, *P* = 0.010), and it alone explained 36.5% of the variation. The residuals of the best models were not spatially autocorrelated (Monte Carlo permutation test, *P* > 0.1). Due to the high collinearity between annual temperature, temperature seasonality and PET (r > 0.8, Additional file 1: Table S3), we conducted an analysis that excluded annual temperature. Of the 64 models constructed, we had the same results as with the analysis that included annual temperature (Additional file 1: Table S4). In addition, when the population from Bangalore was excluded because it had less accurate data (Additional file 1: Table S1), we obtained the same general results as in the analysis that included Bangalore (Additional file 1: Table S5).

**Table 1:** Multiple regression models for Asian common toad body size and environmental predictors

Sex	Predictors in model	r^2^	P	AICc	ΔAICc	W_i_
Male	WD	0.542	0.001	108.7	0	0.433
	WD, Prec.	0.553	0.003	111.0	2.25	0.140
	WD, Temp	0.543	0.004	111.3	2.58	0.119
	WD, AET	0.515	0.005	112.2	3.45	0.077
	WD, P. Seas.	0.503	0.006	112.5	3.82	0.064
Female	P. Seas.	0.338	0.013	125.7	0	0.155
	WD	0.331	0.015	125.8	0.16	0.143
	AET, P. Seas.	0.434	0.013	125.9	0.27	0.135
	WD, Temp	0.410	0.017	126.6	0.89	0.099
	WD, P. Seas.	0.364	0.026	127.7	2.02	0.056
Mean	WD	0.365	0.010	118.1	0	0.271
	WD, Temp	0.399	0.019	119.9	1.77	0.112
	Prec.	0.279	0.025	120.0	1.90	0.105
	WD, Prec.	0.374	0.024	120.5	2.39	0.082
	WD, AET	0.358	0.028	120.9	2.78	0.068

*Temp*. Annual mean temperature, *T. Seas*. Temperature seasonality, *Prec*. Annual total precipitation, *P. Seas*. Precipitation seasonality, *PET* Potential evapotranspiration, *AET* Actual evapotranspiration, *WD* Water deficit. Models are ranked by AICc from the best- to worst-fitting models, and only the top five models are presented

## Discussion

Previous studies of the evolution of anuran body size gave insufficient attention to the ecological traits of different animal clades and focused exclusively on the role of environmental temperature gradients [9–13]. In contrast, we found that in one species of terrestrial anuran living in tropical regions, the key drivers of body size variation were water-related gradients. Different relationships between leading factors in the environment that determine body size in anuran clades may arise due to species-specific ecological traits and the particular environmental background they inhabit [30, 31]. Previous studies on Bergmann’s cline among anurans found a variety of correlations with environmental conditions [3, 4, 12–26, 30–32], and some species showed a coincident cline indicating that temperature seasonality [24] and absolute temperature [13] were the critical factors in the evolution of their body sizes. However, other studies found that precipitation gradients had an important mediating role in the relationship between body size and environment. Interspecific body size variation between anurans in the Brazilian Cerrado was influenced by water deficit level [30], while body size in the male lesser treefrog was influenced by precipitation seasonality [35]. The different environmental backgrounds those species face may result in the diversified clines [30, 45]. On the other hand, many species showed no or a reversed cline along the thermal and precipitation gradients that produce Bergmann’s clines in other species [17–22]. We argue that such patterns may be explained by the fact that previous studies ignored the diverse ecological traits of these species. For instance, aquatic species experience relatively smaller selective pressures from fluctuations in the macroenvironment due to the buffering effect of the aquatic microenvironment [43]. Arboreal and terrestrial species would be expected to respond more to fluctuations in the macroenvironment as they face these conditions directly [24, 42, 43]. Darwin’s frog is a terrestrial frog that lives in temperate forests, and the lesser treefrog is an arboreal species that lives in the tropics. In each species, body size was found to covary with environmental gradients, but in those studies little attention was paid to the important role of their ecologies and environmental backgrounds [24, 35]. Similar patterns and mechanisms also exist among other animal clades. Snakes in Australia with different ecological traits (nocturnal/diurnal activity; fossorial/surface lifestyles) showed different body size-environment relationships. In addition, due to the hot Australian macroenvironment, few areas are cold enough to limit daily activity. Low temperature in other regions [46, 47] does not act on their body size, while high summer temperatures might actually limit their daily activities [48]. Furthermore, some endotherms show the same correlations. The body size of European herbivores showed a weaker relationship with temperature relative to that of carnivores and omnivores [2], and body size in tuco-tucos (*Ctenomys*), a subterranean rodent clade from South America, showed a reversed cline to Bergmann’s rule both between and within species [27]. This is likely due to their subterranean lifestyle, which protects them from external temperature fluctuations and subjects them to other selective pressures, such as precipitation levels driving body size adaptation to different digging conditions [27]. The roles of ecological traits and environmental background seem to be important in the body size-environment relationship in all animal clades. For terrestrial anurans, larger body size in colder climates can help to conserve body heat [31]. Such a mechanism is advantageous in regions where the temperature fluctuates more (i.e., the Holarctic). However, in regions where temperatures fluctuate less, selective pressures from temperature gradients are not strong enough to drive body size diversification [30, 49]. In tropical regions, temperatures are usually high and stable, but precipitation gradients fluctuate. Anurans need to maintain their water balance while still conserving energy [30]. In other words, the key factor influencing the body size-environment relationship among anurans in tropical regions is the dryness level, whereas the temperature gradient is the key factor in temperate regions [30]. The Asian common toad is a terrestrial species that spends the majority of its life on land, except to reproduce. We predict that it will experience greater stress from macroenvironmental gradients than an aquatic species would [31, 43]. Further, this toad’s habitat is characterized by warm and stable ambient temperatures with little fluctuation, but precipitation varies greatly (Additional file 1: Table S2). The conservation of body water because of evaporative water loss becomes a priority, rather than how to conserve heat like terrestrial anurans in other temperate regions [30, 31, 50]. Therefore, body size in the Asian common toad may evolve with changing water deficit gradients [30]. In accordance with our predictions, our results indicated that both male and female body size did not covary with temperature-related parameters, but there was a significant positive correlation between body size and water deficit for both sexes. In addition to the water deficit gradient, the body size of the female Asian common toad showed a positive correlation with precipitation seasonality. It is possible that no linkage between body size and environmental temperature gradient was observed because such mild gradients in temperature and PET do not generate enough selection pressure to drive body size [30, 49]. Our findings were inconsistent with the predictions of the hibernation hypothesis, which emphasizes the role of seasonality in temperature (Additional file 1: Table S4) [24]. Unlike the ambient environment in the habitat of Darwin’s frog, temperature is not strongly seasonal across the Asian common toad’s range [36, 39], which means that the Asian common toad does not experience such drastic seasonal temperature fluctuations and selection pressure would be weak [30, 36, 49]. The Asian common toad is also far larger than Darwin’s frog (species mean body size 73.52 mm vs 23.7 mm), which allows it to reserve more energy to survive the subsequent year after hibernation; in some regions, the Asian common toad does not hibernate at all [51–53]. The hibernation hypothesis may be more applicable to small species living in temperate regions. The water deficit conditions across the distribution of the Asian common toad are similar to fluctuations in water availability in the Brazilian Cerrado [30, 36, 39]. This means that the water deficit gradient is more likely to become a selective pressure on body size [30, 49]. Consistent with our predictions and with the results of the interspecific comparison conducted in the Cerrado [30], the body size of both male and female Asian common toads showed a positive correlation with water deficit gradient; populations in dryer areas are larger-bodied than populations in wetter areas in accordance with the predictions of the water-energy conservation hypothesis [30]. Becoming larger helps to conserve body water in dry environments. Further, in contrast to previous studies [13, 24, 30, 31, 35], we also found a positive correlation between female body size and precipitation seasonality, meaning that female Asian common toads grow larger in regions where there is high seasonality in precipitation. Seasonal changes in precipitation can affect the reproductive strategies of female toads [51–55]. In regions with strong seasonality, total yearly precipitation is concentrated into just a few months [36]. Although these toads spend the majority of life on land, mating and tadpole development must occur in water, typically a temporal pool [56, 57]. Thus, reproductive activity is constrained by the availability of these pools [53–57]. In regions characterized by strong seasonality in precipitation, the window for the toad to mate and for the tadpoles to develop is relatively short. Under such conditions, larger bodies enable female Asian common toads to give birth to as many offspring as possible in a limited time [51]. Where precipitation is more evenly distributed throughout the year, in contrast, every month is suitable for toads to reproduce. For instance, toads have been observed in amplexus every month in Singapore, where the precipitation seasonality index is low [36, 39, 58]. Under such environmental conditions, female Asian common toads are no longer pressured to reproduce in bursts. Similar relationships between body size and seasonality of precipitation were also observed in female *Polypedates leucomystax*, an arboreal species that grows bigger and produces larger eggs in central Thailand than in Singapore. Nevertheless, two co-occurring aquatic species, *Microhyla heymonsi* and *Hylarana erythraea*, collected from the same locations exhibit neither body size nor egg size variation as seen in *P. leucomystax* [59]. The male Asian common toad is not affected by precipitation seasonality in the same way as the female. Differences in the strongest predictors of body size between the sexes may arise due to life history traits, since the body size of each sex is under slightly different selection pressures despite experiencing the same environment [17–22]. Female Asian common toads are larger than males (t = − 6.42, *P* < 0.001; 80.0 mm v.s. 67.0 mm). These larger females suffer relatively less pressure from reduced water availability than males, and as a result female body size is not as tightly coupled to the water deficit gradient [49]. In addition to becoming larger in dry regions to conserve water, female Asian common toads need to lay as many eggs as possible in the short reproductive window such environments allow. As a result, the body size of female toads in regions with highly seasonal precipitation should also increase for that reason. As expected, the body size of male Asian common toads did not vary in response to seasonality of precipitation [60]. The reproductive rhythm of male toads is the same as that of females in the same population [52, 57, 61]. In seasonally breeding populations, female toads must lay as many eggs as possible while they can, which is a costly investment, but sperm production for male toads is far less energy-intensive [60] and therefore would not be expected to affect male body size. These sex-specific reproductive costs are responsible for the different relationships between body size and the precipitation seasonality index observed in males vs. females. In contrast to our findings in the Asian common toad, male lesser treefrogs are bigger in regions with highly seasonal precipitation [35]. Though both terrestrial anurans and arboreal anurans should be more sensitive to the macroenvironment than aquatic anurans [31, 42], there are other ways to conserve heat and energy besides growing larger. Tree frogs typically have thicker, waterproof skin to guarantee their water balance [42], while terrestrial species may adapt behavioral strategies to achieve the same goal [41, 62, 63]. The Asian common toad is larger than lesser treefrogs (species mean body size for males: 67.0 mm v.s. 23.7 mm) [35], which may give them more protection from dry conditions. Therefore, the differences in observed relationships between body size and environmental gradients in the lesser treefrog and the Asian common toad may result from their different lifestyles. More generally, both studies indicated a link between body size and environmental gradients, although the specific factor that explained the variation was dependent on environmental background. The unique responses in body size of male and female toads to environmental gradients suggests that we should take sex into account when analysing intraspecific and interspecific variation, using mean values for each sex as the measurement of species-specific body size [24, 64]. Collapsing the values of male and female body sizes into a single mean for the species may result in information loss; for instance, in the present study, we can detect the correlation between females and seasonality of precipitation gradients only by analyzing the mean SVT for females separately. Our study demonstrated that water deficit plays a key role in geographic body size variation of a tropical terrestrial anuran. In addition, it indicated that precipitation seasonality is another important environmental factor driving body size variation of anurans. Together, the results from studies of the Asian common toad, lesser treefrog and Darwin’s frog suggest that this variation is primarily dependent on the environmental background and ecological traits of different species. For terrestrial anurans in general, water-related gradients act as the main selective pressure in tropical regions, while thermal gradients act as the main pressure in temperate regions [30]. Beyond the role of environmental background and lifestyle, we suggest that species-specific body size itself is an important factor determining geographic variation patterns among anurans. A species’ size is an ecological trait [1, 65, 66], and a relatively larger body size facilitates resistance to harsh environmental conditions, while smaller species are under greater pressure from ambient environmental conditions [65, 66]. For example, a comparative study indicated that body size of lesser treefrog becomes bigger while Blacksmith treefrog (*Boana faber*) enhance its ability of resistance to evaporative water loss with the dehydrating gradient because of their species-specific body size (21.8 mm v.s. 90.8 mm) while enlarge body size for lesser treefrog is more efficient to keep water balance [66]. In the present study, water deficit explains at most 52% of body size variation in male Asian common toads (67.58 mm), while it explains only 31.7% of the variation for females (80.45 mm). However, the seasonality of temperature alone explains 87.6% of the size variation in the much smaller Darwin’s frog (23.7 mm) [24]. As an ecological trait, body size itself will influence the intraspecific body size-environment relationship, with larger species being less sensitive to environmental gradients. It should be noted that all data in this study were summarized from the literature. Since the measurements were conducted by different people (Additional file 1: Table S1), there may be some errors in the dataset. Thus, further study in the field or laboratory will be required to replicate these results. Another limitation is that we explained at most only half of the variation with environmental factors, which means that more than half of the observed patterns are not associated with local climate. Size-independent traits such as behavioral [62] and psychological [63] strategies or different waterproof ability [42] or interaction of those strategies [66] are employed to survive under shifting environmental conditions. Besides, many previous studies on intraspecific size variation of anurans indicated that life history traits such as growth rate, longevity, age at sexual maturity, clutch size and egg size may all contribute to geographic body size variation and many authors concerned more on the effects of those traits on the anuran body size variation [3, 4, 14–19]. However, those intrinsic traits also covary with different selective pressures like body size [3, 4, 16, 17, 67]. The life history traits of different geographical populations represent interaction and trade-offs under particular environmental conditions and are not fully separable from body size, and body size is the representation of those life history traits evolve and interact with each other [3, 4, 16, 67]. Thus, it is improper to emphasize and conclude that those life history traits alone can produce the observed geographic patterns in body size. On the contrary, it is reasonable to explore how body size (the outcome of all other life history traits evolve and interact with each other along different environmental gradients) evolves under the ambient environments. Similar to the Bergmann’s rule [9, 26, 33], though many intraspecific studies have found some general trends in the diversification of life history traits [67], many species do not show the same trends [19, 20, 44, 68], it is more interesting to explore why some species do not show the same cline with the general trend. Under the framework of intraspecific Bergmann’s rule, it will gain better insight into how body size evolves if we consider how different life history traits evolve and interact with each other under the same selective pressures and how many of them contribute to the size variation alone [69]. Furthermore, interaction with sympatric species in the community is also a driving force act on the size variation of animals [5, 6], however, due to the limitations of our method, we could not account for those life history traits and relationship of the toad with other species in the community in this study. Further studies conducted in the field or laboratory could examine the evolution of those life history traits under environmental gradients and their relationship with body size in greater detail [44, 67–69]. Besides, accounting for issues such as interaction with sympatric species [5, 6], time spent in hibernation [50, 51, 54, 70, 71] or behavioral [62] and physiological [63] adaptation is also required to gain insight into all of the potential mechanisms driving body size evolution not only in the Asian common toad, but in anurans more generally, across their various habitats.

## Conclusions

Using the Asian common toad as a focal species, our study demonstrated that at the intraspecific level, body size variation of terrestrial anurans in the Asian tropics is primarily determined by water-related, rather than temperature-related, environmental parameters, a pattern consistent with the predictions of the water-energy conservation hypothesis. Additionally, we documented that even in the same environmental conditions, females and males of the same species may evolve different adaptive strategies. Combined with findings in Darwin’s frog and the lesser treefrog, our results suggest that selective pressures at the intraspecific level differ with changes in the macroenvironment.

## Methods

### Species data and environmental predictors

Snout-vent length (SVL) was used to measure body size in the Asian common toad. We summarized data on SVL and the central coordinates of each geographic population from the published literature (published research articles and local faunas: Additional file 1: Table S1). Because our goal was to explore whether the body size of different geographic populations covaries with particular environmental gradients, the mean (mean ± SD) SVL of mature individuals of each population was used as the measure of central tendency [24, 72]. Though previous studies indicated that life history traits such as age and growth rate also contribute to geographic variability in body size [67], those traits also evolve under different selective pressures with interaction and trade off exist among them [3]. In other words, the life history traits of different populations trade off because of environmental gradients and can themselves be represented in the form of body size diversification [3, 4, 16, 19]. Since male and female amphibians may respond differently to climatic variation [17, 18, 20, 21], analyses performed on unsexed populations can be biased [72]; we employed the mean body size of both sexes together as the estimate of the population’s body size and separately to conduct our analysis. Furthermore, to better compare with the study of Darwin’s frogs [24] and interspecific studies [13, 30, 64], we conducted an extra analysis of unsexed mean SVL by population. Because body size evolves under direct selection resulting from different environmental pressures, analyses linking geographic parameters with body size probably cannot capture the causal forces behind the observed patterns [28, 32]. In the present study, we used several environmental gradients in lieu of examining toad body size variation by latitude or altitude. Following previous studies [13, 23, 24, 30, 35], seven environmental factors that may serve as evolutionary determinants of anuran body size were selected and extracted according to the geographic coordinates of the sampled populations that were collected (Additional file 1: Table S1 and Table S2). Then, the relationship between these environmental factors and the body size of Asian common toads was analysed to determine whether they work as selective pressures on the evolution of toad body size. The seven factors are: annual mean temperature (°C), a measure of heat in the environment; annual total precipitation (mm), a measure of water availability; temperature seasonality (standard deviation of temperature across months), an indicator of energy predictability; precipitation seasonality (coefficient of variation of precipitation across months), an indicator of water predictability; actual evapotranspiration (AET, mm), a measure of water and energy balance; potential evapotranspiration (PET, mm), a measure of heat and light inputs; and the water deficit (WD, mm), a measure of dryness level. Data on annual mean temperature, annual total precipitation, temperature seasonality and precipitation seasonality were extracted from WorldClim at a resolution of 0.167° × 0.167° grid cells [36]. Data on actual evapotranspiration and potential evapotranspiration were extracted according to Willmott & Matsuura (2001) at a resolution of 0.5° × 0.5° grid cell [37]. Water deficits were calculated by subtracting AET from PET [30, 37, 38].

### Statistical analyses

Ordinary least squares regression based on Akaike’s information criterion (AIC) was used to generate multiple regression models, with body size as the response value and environmental variables as predictors. We compared the AICc scores of models with all possible predictor combinations. The model with the lowest score was selected as the final, best-fitting model, thus omitting uninformative predictors [73]. Due to the high collinearity between annual temperature and temperature seasonality/PET (r > 0.8, Additional file 1: Table S3), we excluded temperature seasonality and PET in the final analysis. As a check, we also conducted an analysis including temperature seasonality and PET but excluding annual temperature, which produced similar results (Additional file 1: Table S4). Two-tailed significance levels were set at *P* = 0.05. Multiple regression was run in R (version 3.1.1-R Core Team 2015) [74] using the package ‘MuMIn’ [75]. ARCGIS 10.0 was used to calculate Moran’s *I* as a measure of spatial autocorrelation of SVL [76], and the Monte Carlo permutation test (199 permutations) was used in SAM 3.0 to assess spatial autocorrelation of residuals [77].

## Supplementary information

**Additional file 1: Table S1.** Mean body size data of Asian common toad from sampled populations across its distribution range (ranked from north to south). **Table S2.** Data of environmental predictors of sampled populations. **Table S3.** Correlation coefficients between each environmental variable (statistically significant [*P* < 0.05] are shown in bold). **Table S4.** Multiple regression models for mean body size of each sex of Asian common toad and environmental predictors (excluding annual mean temperature due to it is highly correlated with temperature seasonality and potential evapotranspiration). Models are ranked by AICc from the best- to worst-fitting models. **Table S5.** Multiple regression models for mean body size of each sex of Asian common toad and environmental predictors (excluding temperature seasonality and potential evapotranspiration due to they are highly correlated with annual mean temperature), excluding the population from Bangalore due to its less accurate data. Models are ranked by AICc from the best- to worst-fitting models

## Data Availability

The datasets used and analysed during the current study are available in additional file 1 Table S1 and Table S2.

## Abbreviations

AET: Actual evapotranspiration
AIC: Akaike’s information criterion
P. Seas: Precipitation seasonality
PET: Potential evapotranspiration
Prec: Annual total precipitation
SVL: Snout-vent length
T. Seas: Temperature seasonality
Temp: Annual mean temperature
WD: Water deficit
