# Supplementary material for: Geographic body size variation of a tropical anuran: effects of water deficit and precipitation seasonality on Asian common toad from southern Asia
Source: BMC Evol Biol. 2019 Nov 9;19:208. doi: 10.1186/s12862-019-1531-z (PMC6842474; doi:10.1186/s12862-019-1531-z)
Supplement: Supplementary file 1 — Additional file 1: Table S1. Mean body size data of Asian common toad from sampled populations across its distribution range (ranked from north to south). Table S2. Data of environmental predictors of sampled populations. Table S3. Correlation coefficients between each environmental variable (statistically significant [P < 0.05] are shown in bold). Table S4. Multiple regression models for mean body size of each sex of Asian common toad and environmental predictors (excluding annual mean temperature due to it is highly correlated with temperature seasonality and potential evapotranspiration). Models are ranked by AICc from the best- to worst-fitting models. Table S5. Multiple regression models for mean body size of each sex of Asian common toad and environmental predictors (excluding temperature seasonality and potential evapotranspiration due to they are highly correlated with annual mean temperature), excluding the population from Bangalore due to its less accurate data. Models are ranked by AICc from the best- to worst-fitting models [file 12862_2019_1531_MOESM1_ESM.docx]

**Table S1.** Mean body size data of Asian common toad from sampled populations across its distribution range (ranked from north to south).

| Population | Longitude  (°) | Latitude  (°) | **Female** **(mm)** | N | Min  (mm) | Max  (mm) | **Male**  **(mm)** | N | Min  (mm) | Max  (mm) | **Mean**  **(mm)** |
| --- | --- | --- | --- | --- | --- | --- | --- | --- | --- | --- | --- |
| Lishui [1] | 119.9 | 28.5 | 56.9 | 150 | 41.3 | 69.4 | 50.8 | 149 | 39.8 | 63.7 | 53.6 |
| Wuyanling [2] | 119.8 | 27.8 | 72.6 | 45 | 54.0 | 90.0 | 58.9 | 45 | 48.1 | 72.7 | 65.2 |
| Guilin [3] | 110.3 | 25.3 | 90.2 | 7 | 85.0 | 97.0 | 69.7 | 10 | 58.5 | 79.0 | 79.9 |
| Shaoguan [1] | 113.5 | 24.8 | 62.4 | 14 | 53.3 | 74.5 | 59.0 | 27 | 50.2 | 76.3 | 63.6 |
| Jingdong [4] | 100.9 | 24.5 | 103.5 | 10 | 95 | 112 | 76.5 | 10 | 72 | 81 | 90.0 |
| Taizhong [5] | 120.6 | 24.2 | 73.4 | 144 | N/A^a^ | N/A | 59.7 | 301 | N/A | N/A | 66.6 |
| Zhaoqin [1] | 112.5 | 23.1 | 61.2 | 73 | 46.2 | 82.8 | 50.7 | 32 | 48.6 | 67.1 | 61.2 |
| Bhubaneswar [6] | 85.8 | 20.3 | 79.1 | 20 | 46.5 | 104.0 | 67.2 | 18 | 41.5 | 78.0 | 67.5 |
| Pune [7] | 73.9 | 18.5 | 89.7 | 19 | 62.8 | 118.1 | 71.0 | 39 | 52.8 | 82.9 | 79.2 |
| Qiongzhou [1] | 109.7 | 18.2 | 77.8 | 201 | 44.8 | 109.9 | 60.5 | 122 | 47.3 | 75.1 | 69.3 |
| Bach Ma [8] | 107.8 | 16.1 | 81.3 | 56 | 65.2 | 97.3 | 60.7 | 53 | 50.2 | 70.3 | 70.8 |
| Dharwad [9] | 75.1 | 15.5 | 107.1 | 148 | 88.0 | 123.0 | 90.6 | 148 | 72.5 | 110.5 | 98.5 |
| Bangkok [10] | 100.6 | 13.7 | 91.1 | 41 | 68.0 | 108.0 | 81.3 | 27 | 65.0 | 92.0 | 86.2 |
| Bangalore [11] | 77.6 | 12.9 | 94.5^b^ | N/A | N/A | N/A | 79.1^c^ | N/A | N/A | N/A | 88.9 |
| Banjuwangi [12] | 114.4 | -8.2 | 59.0 | 109 | 41.0 | 86.0 | 61.0 | 59 | 44.0 | 77.0 | 62.0 |

^a^ Indicates that there is no data available

^b^ Transferred according to body mass (body weight [g] = 0.1 SVL [mm]^3^)

^c^ Inferred according to female SVL and mean sexual dimorphism ratio of other 14 populations

**References**

1. Fan XL. Thermal adaption of *Duttaphrynus melanostictus*: pattern and mechanism. Ph. D. Thesis. Nanjing: Nanjing normal university; 2014. (in Chinese)
2. Zheng FD, Wang YW, Liu X, Li SR, Zhang YP. Sexual dimorphism and assortative mating pattern in Duttaphrynus melanostictus. Chin J of Zool. 2018;53:360–367. (in Chinese)
3. Zhang YX, Wen YT. Amphibians in Guangxi. Guilin: Guangxi Normal University Press; 1997. (in Chinese)
4. Fei L, Ye C. Amphibians of China (volume I). Beijing: Science Press; 2016.
5. Shieh JN. The breeding ecology of *Bufo melanostitus*. M.S. Thesis. Taiwan, ROC: Tunghai University; 1993. (in Chinese)
6. Nayak S, Mahapatra PK, Mohanty RK, Dutta SK. Age determination by skeletochronology in the common Indian toad *Bufo melanostictus* SCHNEIDER, 1799 (Anura: Bufonidae). Herpetozoa. 2007;19:111–119.
7. Narayan EJ, Gramapurohit NO. Sexual dimorphism in baseline urinary corticosterone metabolites and their association with body-condition indices in a peri-urban population of the common Asian toad (*Duttaphrynus melanostictus*). Comp Biochem Phys A. 2016;191:174–179.
8. Ngo BV, Ngo CD. Reproductive activity and advertisement calls of the Asian common toad *Duttaphrynus melanostictus* (Amphibia, Anura, Bufonidae) from Bach Ma National Park, Vietnam. Zool Stud. 2013;52:12.
9. Jorgensen CB, Shakuntala K, Vijayakumar S. Body size, reproduction and growth in a tropical toad, *Bufo melanostictus*, with a comparison of ovarian cycles in tropical and temperate zone anurans. Oikos. 1986;46:379–382.
10. Alexander G. Bidder's organ in *Bufo melanostictus* Schneider. Copeia. 1932;1932:78–80.
11. Gramapurohit NP, Radder RS. Mating pattern, spawning behavior, and sexual size dimorphism in the tropical toad *Bufo melanostictus* (Schn.) J Herpetol. 2012;46:412–416.
12. Church G. The Invasion of Bali by *Bufo melanostictus*. Herpetologica. 1960;16:15–21.

**Table S2.** Data of environmental predictors of sampled populations.

| Population | Temp. (℃) [1] | T. Seas. [1] | Prec. (mm) [1] | P. Seas. [1] | PET (mm) [2] | AET (mm) [2] | WD (mm) [2,3] |
| --- | --- | --- | --- | --- | --- | --- | --- |
| Lishui | 17.9 | 7793 | 1492 | 49 | 62.75 | 62.29 | 0.46 |
| Wuyanling | 15.8 | 7065 | 1804 | 49 | 63.052 | 62.77 | 0.282 |
| Guilin | 19.5 | 7205 | 1771 | 66 | 84.19 | 80.74 | 3.45 |
| Shaoguan | 20.4 | 6857 | 1517 | 61 | 92.72 | 84.42 | 8.30 |
| Jingdong | 17.5 | 3960 | 1017 | 85 | 74.44 | 70.10 | 4.34 |
| Taizhong | 22.3 | 4419 | 1687 | 84 | 94.74 | 88.27 | 6.47 |
| Zhaoqin | 22.1 | 5502 | 1631 | 65 | 90.45 | 88.33 | 2.12 |
| Bhubaneswar | 27.3 | 3214 | 1508 | 100 | 139.48 | 91.13 | 48.35 |
| Pune | 25.0 | 2624 | 768 | 104 | 109.5 | 62.95 | 46.55 |
| Qiongzhou | 24.0 | 3208 | 1351 | 81 | 113.53 | 88.8 | 24.73 |
| Bach Ma | 24.6 | 2909 | 2353 | 74 | 94.74 | 85.47 | 9.27 |
| Dharwad | 24.7 | 1947 | 792 | 79 | 120.28 | 54 | 66.28 |
| Bangkok | 28.1 | 1310 | 1455 | 82 | 151.22 | 105.88 | 45.34 |
| Bangalore | 23.8 | 2045 | 842 | 82 | 104.87 | 63.57 | 41.30 |
| Banjuwangi | 26.2 | 844 | 1444 | 49 | 105.54 | 102.16 | 3.38 |

**References**

1. Hijmans RJ, Cameron SE, Parra JL, Jones PG, Jarvis A. Very high resolution interpolated climate surfaces for global land areas. International J Climatol. 2005;25:1965–78.
2. Willmott CJ, Matsuura K. Terrestrial water budget data archive: monthly time series (1950–1999). 2001. Version, 1.02. Accessed 30 May 2013.
3. Francis AP, Curri, DJA. globally-consistent richness-climate relationship for angiosperms. Am Nat. 2003;161;523–36.

**Table S3.** correlation coefficients between each environmental variable (statistically significant [P < 0.05] are shown in bold).

| Parameters | Temp. | T. Seas. | Prec. | P. Seas. | PET | AET | WD |
| --- | --- | --- | --- | --- | --- | --- | --- |
| Temp. | 1 |  |  |  |  |  |  |
| T. Seas. | **-0.827** | 1 |  |  |  |  |  |
| Prec. | 0.157 | 0.389 | 1 |  |  |  |  |
| P. Seas. | **0.518** | -0.502 | -0.413 | 1 |  |  |  |
| PET | **0.920** | **-0.740** | -0.254 | **0.601** | 1 |  |  |
| AET | 0.507 | -0.276 | 0.469 | -0.016 | 0.468 | 1 |  |
| WD | **0.669** | **-0.630** | **-0.613** | **0.681** | **0.784** | -0.181 | 1 |

Temp., Annual mean temperature; T. Seas., Temperature seasonality; Prec., Annual total precipitation; P. Seas., Precipitation seasonality; PET, Potential evapotranspiration; AET, actual evapotranspiration; WD, water deficit

**Table S4.** Multiple regression models for mean body size of each sex of Asian common toad and environmental predictors (excluding annual mean temperature due to it is highly correlated with temperature seasonality and potential evapotranspiration). Models are ranked by AICc from the best- to worst-fitting models.

| Sex | Predictors in model | r^2^ | P | AICc | ΔAICc | W_i_ |
| --- | --- | --- | --- | --- | --- | --- |
| Male | WD | 0.542 | 0.001 | 108.7 | 0 | 0.324 |
|  | WD, Prec. | 0.553 | 0.003 | 111.0 | 2.25 | 0.105 |
|  | WD, T. Seas. | 0.523 | 0.004 | 111.9 | 3.21 | 0.065 |
|  | AET, PET | 0.516 | 0.005 | 112.2 | 3.45 | 0.058 |
|  | WD, AET | 0.515 | 0.005 | 112.2 | 3.45 | 0.058 |
| Female | P. Seas. | 0.338 | 0.013 | 125.7 | 0 | 0.131 |
|  | WD | 0.331 | 0.015 | 125.8 | 0.16 | 0.121 |
|  | AET, P. Seas. | 0.434 | 0.013 | 125.9 | 0.27 | 0.114 |
|  | WD, P. Seas. | 0.346 | 0.026 | 127.2 | 2.02 | 0.048 |
|  | Prec., P. Seas. | 0.360 | 0.027 | 127.8 | 2.11 | 0.045 |
| Mean | WD | 0.365 | 0.010 | 118.1 | 0 | 0.195 |
|  | Prec. | 0.279 | 0.025 | 120.0 | 1.90 | 0.075 |
|  | WD, Prec. | 0.374 | 0.024 | 120.5 | 2.39 | 0.059 |
|  | AET, T. Seas. | 0.373 | 0.024 | 120.5 | 2.49 | 0.058 |
|  | WD, PET | 0.373 | 0.024 | 120.9 | 2.77 | 0.049 |

**Table S5.** Multiple regression models for mean body size of each sex of Asian common toad and environmental predictors (excluding temperature seasonality and potential evapotranspiration due to they are highly correlated with annual mean temperature), excluding the population from Bangalore due to its less accurate data. Models are ranked by AICc from the best- to worst-fitting models.

| Sex | Predictors in model | r^2^ | P | AICc | ΔAICc | W_i_ |
| --- | --- | --- | --- | --- | --- | --- |
| Male | WD | 0.508 | 0.002 | 102.7 | 0 | 0.482 |
|  | WD, Prec, | 0.504 | 0.008 | 105.6 | 2.92 | 0.112 |
| Female | P. Seas. | 0.324 | 0.019 | 118.3 | 0 | 0.212 |
|  | WD | 0.293 | 0.026 | 118.9 | 0.63 | 0.155 |
|  | AET, P. Seas. | 0.395 | 0.025 | 119.6 | 1.27 | 0.113 |
|  | WD, Temp | 0.367 | 0.032 | 120.2 | 1.91 | 0.082 |
|  | WD, P. Seas. | 0.329 | 0.044 | 121.0 | 2.72 | 0.055 |
| Mean | WD | 0.322 | 0.020 | 111.5 | 0 | 0.287 |
|  | Prec. | 0.215 | 0.054 | 113.6 | 2.07 | 0.102 |
